# Case report: Presumptive subcutaneous malignant peripheral nerve sheath tumor with intracranial invasion and osteolysis in the posterior fossa of a dog

**DOI:** 10.3389/fvets.2022.977099

**Published:** 2022-11-08

**Authors:** Kyosuke Hidari, Yuya Nakamoto, Keiichi Sakurai, Yoko Sakurai, Kazumi Nibe, Miwa Nakamoto

**Affiliations:** ^1^Neuro Vets Animal Neurology Clinic, Kyoto, Japan; ^2^Veterinary Medical Center, Osaka Metropolitan University, Osaka, Japan; ^3^Sakura Animal Hospital, Kyoto, Japan; ^4^FUJIFUILM VET Systems Co., Ltd., Tokyo, Japan

**Keywords:** peripheral nerve sheath tumor, extracranial tumor, intracranial invasion, malignant lesion, osteolysis, occipital region

## Abstract

A 13-year-old castrated male Toy Poodle presented with an acute vestibular disorder. Magnetic resonance imaging and computed tomography revealed a large oval space-occupying mass with skull destruction located from the subcutaneous tissue to the posterior fossa region. Histopathologically, the mass was a bundled growth of spindle-shaped mesenchymal tumor cells between the myofibrillar and collagen bundles. The cells were moderately irregular in size and had eosinophilic stained cytoplasm. The cells were highly atypical and had rare mitotic figures. Neoplastic cells were immunoreactive for S100, GFAP, Olig-2, SOX10 and immunonegative for NF, E-cadherin, and Claudin-1. Collective findings were presumptive with a diagnosis of malignant peripheral nerve sheath tumor.

## Introduction

Peripheral nerve sheath tumors (PNSTs) mainly occur in the sheaths of peripheral nerves, among which malignant PNSTs (MPNSTs) have a poor prognosis. In MPNSTs, distant metastasis is uncommon, while local invasiveness is high. In addition to the peripheral nerves, PNSTs in dogs are derived from the central nervous system (CNS) and various other sites (1), including the greater omentum, adrenal gland, and third eyelid (2–4), with clinical signs and prognosis differing according to the site. MPNSTs have been reported in the intracranial region (5–7).

## Case description

**Figure 1:**
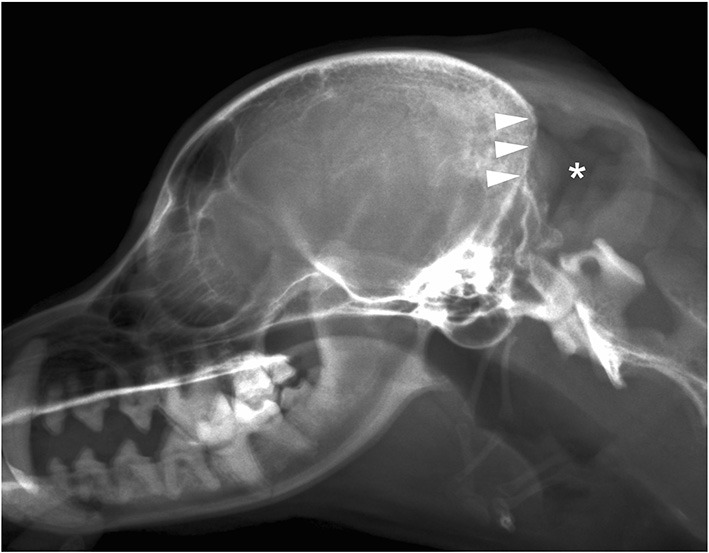
Plain X-ray film of the lateral view of the head. Radiolucent mass shadows in the occipital region (*) and opacification of the occipital bone (arrowhead) were observed.

**Figure 2:**
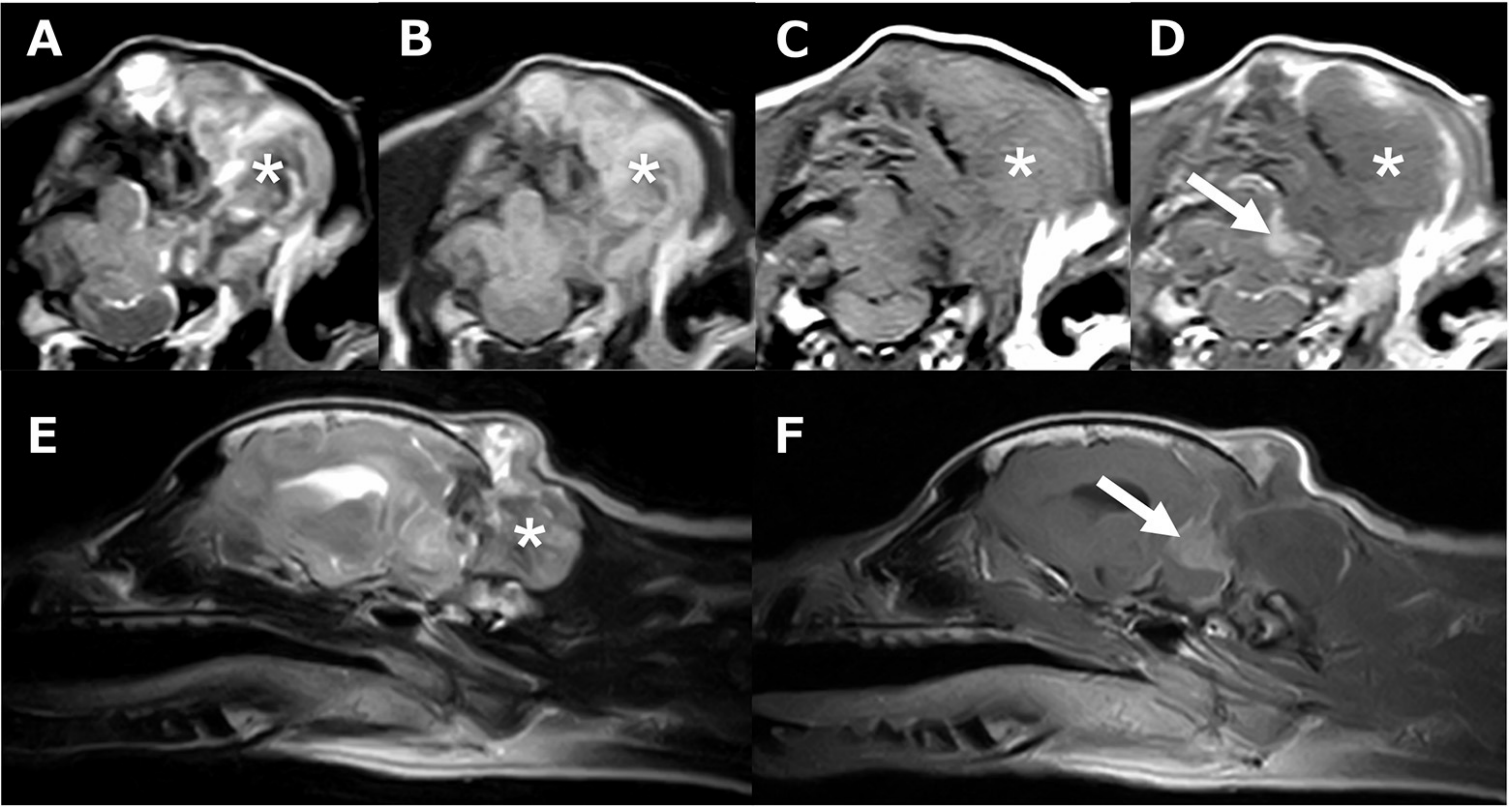
Head MR images. Transverse T2 weighted **(A)**, FLAIR **(B)**, T1 weighted **(C)**, and T1 weighted post-contrast **(D)** images at the level of posterior fossa, and sagittal T2 weighted **(E)** and T1 weighted post-contrast **(F)** at the level of the left cerebellum. A large mass lesion was found in the left occipital subcutaneous tissue, posterior fossa, and left cerebellar bridge angle region. The mass showed mixed hyper intensity was recognized extracranial region (*) and strong enhancement on the margins and near the meninges (arrow).

**Figure 3:**
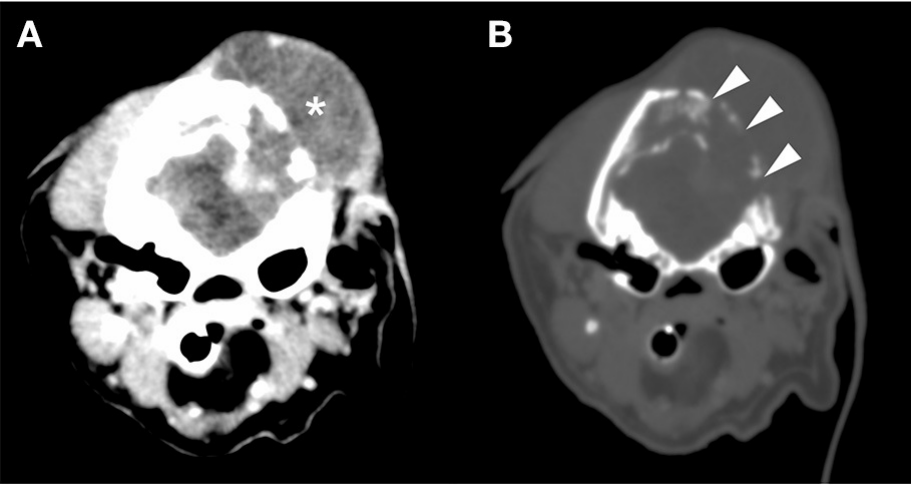
Cranial CT images [**(A)** bone conditions, **(B)** soft tissue conditions]. Contrast-enhanced CT image of the head. Soft tissue mass formation was observed mainly in the left temporal-occipital region (*). Furthermore, the region showed destruction of the occipital bone (arrowhead) and an unclear boundary with the brain parenchyma. No obvious metastatic findings were found in the chest or abdomen. CT, computed tomography.

**Figure 4:**
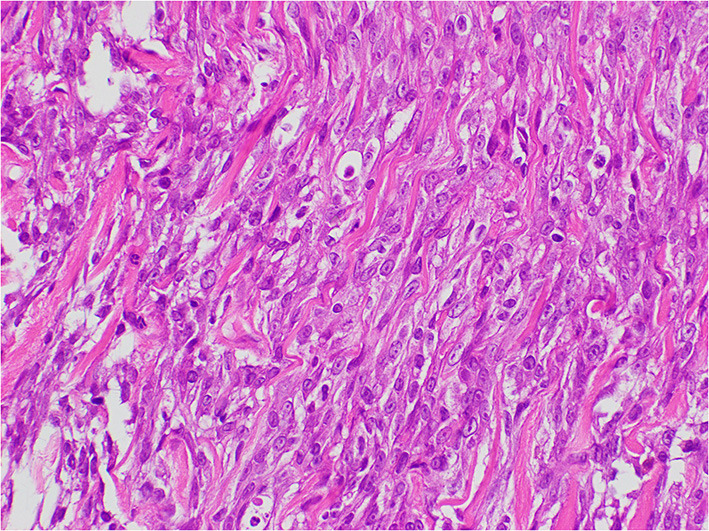
Histopathology of the tumor lesions. A bundle-like growth of spindle-shaped mesenchymal tumor cells was observed between the myofibril and the collagen bundles. The tumor cells were moderately irregular in size and had eosinophilically stained cytoplasm. The tumor cells were highly atypical, and mitotic figures were rare. No intravascular invasion of the tumor in the search area was detected.

**Figure 5:**
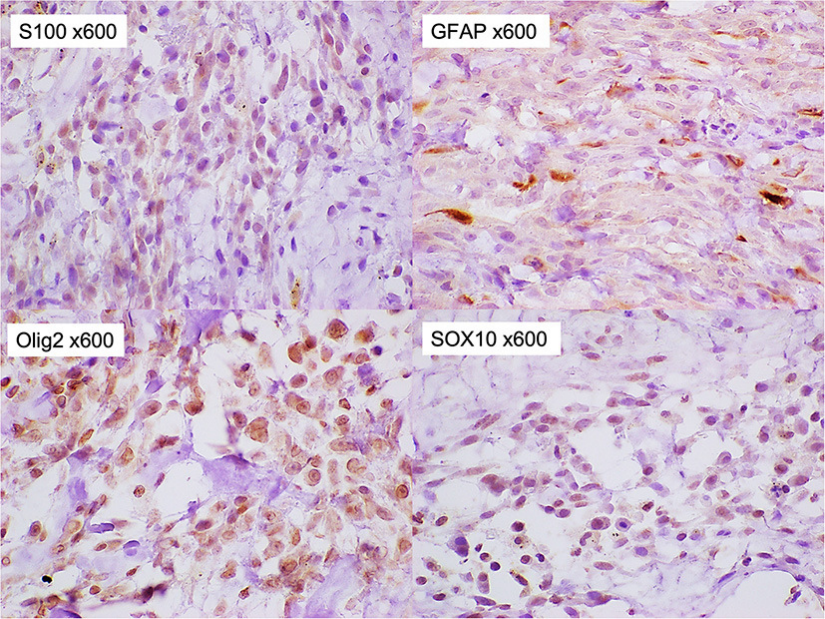
Immunohistochemical staining of the tumor. The tumor cells were stained positively for S-100, glial fibrillary acidic proteins, Olig-2, and SOX10 (x 600).

**Figure 6:**
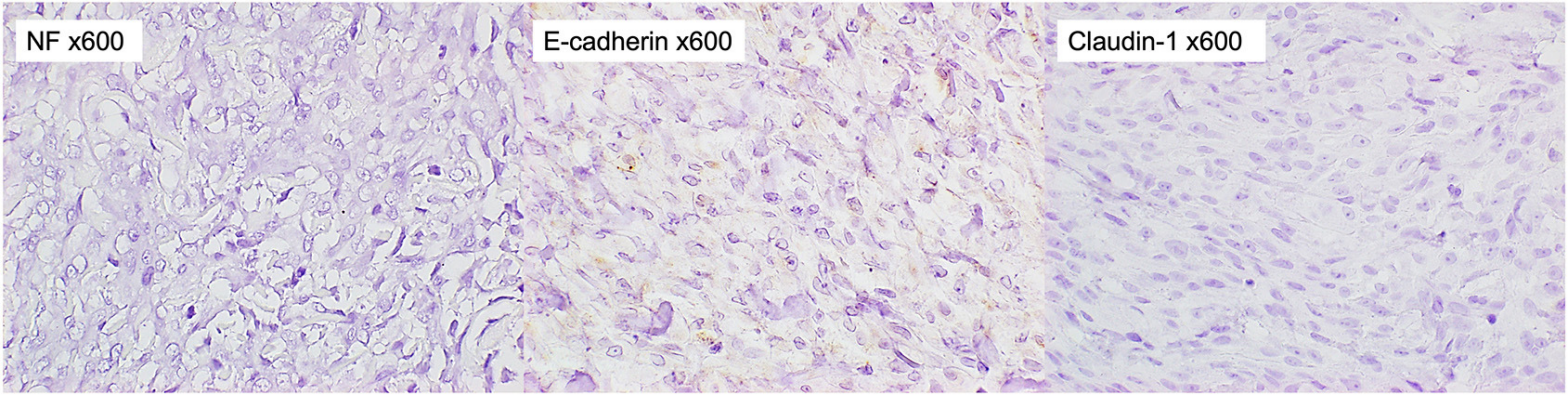
Immunohistochemical staining of the tumor. The tumor cells were stained negatively for NF, E-cadherin, and Claudin-1 (x 600).

A 13-year-old, 5.0-kg castrated male Toy Poodle developed acute vestibular disorder, including horizontal nystagmus and stagger (day 1). Complete blood count results were normal at the referring hospital, whereas serum biochemical examinations showed a slight increase in alkaline phosphatase (160 U/L, reference range: 0–89 U/L) and blood urea nitrogen (36.0 mg/dL, reference range: 9.2–29.2 mg/dL) levels. Six days later, the patient was referred to our hospital because of worsening clinical signs (day 7). On admission, physical examination revealed a body temperature of 39.0°C, a pulse rate of 100 beats/min, panting, and a subcutaneous mass in the left occipital region. A neurological examination revealed ataxia with hypermetria, right-sided head tilt, left-sided hemiparesis, and induction of vertical nystagmus in the dorsal position. Except for disorder to the vestibular area, no other obvious cranial nerve deficits were noted. Mental status was alert. Based on these findings, paradoxical vestibular disorder was suggested, and the left cerebellar lesion was suspected. Differential diseases considered included cerebellar infarction, brain tumor, and meningoencephalitis. Head radiographs showed a mass with increased internal permeability and bone obscuration in the occipital area (Figure 1); however, the thoracic radiographs were unremarkable. Based on these findings, brain magnetic resonance imaging (MRI) and head computed tomography (CT) were recommended. Brain MRI was performed on admission, under general anesthesia, using a 0.4-T MRI system with a permanent magnet (APERTO Lucent, Hitachi, Tokyo, Japan), with the patient in the prone position. We used a human wrist coil and obtained T2-weighted images (T2WI: fast spin echo [FSE], repetition time/echo time [TR/TE] = 4,375/100), fluid-attenuated inversion recovery (FLAIR: fast inversion recovery [FIR], TR/TE = 8,292/78), and T1-weighted images (T1WI: spin-echo [SE], TR/TE = 400/15) with and without intravenous (IV) gadodiamide (0.2 mL/kg, OMNISCAN, GE Healthcare Pharma, Tokyo, Japan) in the transverse, sagittal, and dorsal planes. The MRI findings demonstrated a large oval space-occupying mass located from the subcutaneous tissue in the left occipital region to the posterior fossa and left cerebellar bridge angle region. The mass showed mixed hyperintensity on T2WI and FLAIR, mild hypointensity on T1WI, and strong enhancement on the margins and near the meninges on post-contrast T1WI (Figure 2). Additionally, MRI revealed brain edema around the mass, suggesting increased intracranial pressure. Head CT conducted using a CT scanner (Aquilion Lightning/Helios i Edition, Canon Medical Systems, Tochigi, Japan) showed occipital bone destruction at the lesion site (Figure 3). Since no obvious abnormal findings were found on chest and abdominal CT, the head mass was considered the primary lesion. Tru-cut biopsy was then performed to identify the lesion. Histopathological examination revealed bundled growth of spindle-shaped mesenchymal tumor cells between the myofibrillar and collagen bundles. The tumor cells were moderately irregular in size and had eosinophilic stained cytoplasm. The cells were highly atypical and had rare mitotic figures, whereas no intravascular tumor invasion was observed within the search area (Figure 4). For the immunohistochemical evaluation of the neoplastic cells, some antibodies below, antiglial fibrillary acidic protein (GFAP), anti-s100 protein, anti-neurofilament (NF), anti-olig-2, anti-sox10, anti-e-cadherin, and anti-claudin-1, were used. Neoplastic cells were positive for S100, GFAP, Olig-2, and SOX10 (Figure 5). However, they were negative for NF, E-cadherin, and Claudin-1 (Figure 6). The list of primary antibodies and protocols used in this study were summarized in Table 1. Based on the results of immunohistochemical staining, a diagnosis of suspected MPNST was made.

**Table 1:** Primary antibodies and protocols for immunohistochemistry.

**Antibody**	**Type**	**Dilution**	**Source**	**Antigen retrieval**
S100	pAb	1:500	Dako	-
Olig2	pAb	1:500	Millipore	HIER (Tris-EDTA, pH9.0), 121°C, 10min
SOX10	mAb(1E6)	1:50	Sigma	-
GFAP	pAb	1:500	Dako	-
NF	mAb(2F11)	1:250	CELLMARQUE	-
E-cadherin	mAb(4A2C7)	1:1000	BD pharmingen	HIER (Citrate buffer, pH6.0), 121°C, 10min
Claudin	pAb	1:500	Abcam	HIER (Citrate buffer, pH6.0), 121°C, 10min

mAb, monoclonal antibody; pAb, polyclonal antibody; HIER; Heat-induced epitope retrieval.

Palliative therapy was administered at the owner's request. From day 7, prednisolone (prednisolone tablets; Takeda; 1 mg/kg once a day) and isosorbide (ISOBIDE; Kowa; 1 mg/kg twice a day) were started to reduce cerebral edema and intracranial pressure. On day 11, the patient exhibited intermittent tachypnea, with no obvious improvement in clinical signs. Mental status was depressed mildly, however, palpebral reflex or gag reflex were normal. Prednisolone was then changed to 0.5 mg/kg twice a day along the owner's wishes, but he was unable to stand on day 18. Upon arrival at the hospital, decreased consciousness, decerebellate rigidity, and tachypnea were observed, suggesting severe intracranial hypertension. After treatment for decreasing intracranial pressure (mannitol 2 g/kg constant rate infusion for 30 min, furosemide 1 mg/kg IV), his conscious level recovered, his respiratory state stabilized, and he was discharged. On day 19, his condition deteriorated, and the patient died.

## Discussion

In the present case, the mass lesion extended intracranially and extracranially. Whether the tumor invaded from the outside in or the inside out had not been definitively determined, since postmortem examination was not available. At the onset of neurological symptoms, a large extracranial mass was already present, with destruction of the skull. Based on the time lapse from onset to diagnosis, it was considered highly likely that the tumor originated in the extracranial subcutaneous region and invaded intracranially with skull destruction.

Meningioma is the most common primary intracranial tumor in dogs, followed by glioma and histiocytic sarcoma (8). However, the formation of extracranial lesions with osteolysis has neither been reported in these tumors nor been reported in other primary intracranial tumors. A dog with glioma accompanied by temporal bone erosion has been reported, but no obvious extracranial lesion formation was observed (9). In humans, only rare cases of intracranial gliomas forming extracranial lesions with bone erosion have been reported (10, 11). Thus, it is extremely rare for primary intracranial tumors to spread extracranially with osteolysis in dogs. On the other hand, MPNSTs with bone erosion have been reported in dogs and cats (12, 13). In addition, primary scalp MPNSTs with cranial destruction and intracranial extension have been reported in humans (14). Rhabdomyosarcoma should also be carefully differentiated as a bone-destroying tumor arising in the head and neck region (15). From these reports, the possibility that the extracranial subcutaneous tumor in the occipital region could have caused intracranial invasion was considered in the patient.

In the immunochemical staining of timestamp performed in this study, almost all tumor cells were positive for SOX10 and Olig-2. A few of them were also positive for S100 and GFAP. On the other hand, they were negative for NF, E-cadherin, and Claudin-1. Among these staining results, positive results for Olig-2 and GFAP supported glioma as an important differential (16). However, positive results for S100 and SOX10, which have been reported to be highly specific for MPNST (17–19), were considered negative for glioma. In addition, the strong positive finding of Olig-2 further supported MPNSTs of peripheral nerve origin and ruled out perivascular wall tumors, which are considered to have very similar histopathologic features to MPNSTs (1, 20). From these findings, the tumor in this case was concluded the subcutaneous MPNST. E-cadherin plays an important role in morphogenesis and is involved in invasion and metastasis of tumor tissues (21, 22). The negative E-cadherin result suggested that the MPNST in this case was more likely to be invasive. In our study, however, the small sample size of the tumor tissue collected limited the antibodies that could be used in the immunochemical staining. Laminin in nerve fibers is produced by Schwann cells and is present on the axon surface, basolateral membrane, and in Schwann cells (23). A positive laminin result would further confirm that the tumor was of peripheral nerve origin. Alpha-smooth muscle actin (α-SMA) is the actin isoform that predominates within vascular smooth-muscle cells (24). Myogenin and MydoD1 are included in several transcription factors that regulate the differentiation of skeletal muscle (25). Rhabdomyosarcoma was excluded if α-SMA and skeletal muscle markers, myogenin or MydoD1, were negative.

Most MPNSTs in the cranial nerve show hyperintensity on T2WI and FLAIR, hypo- or iso-intensity on T1WI, and a homogeneous enhancement effect of contrast media on MRI (26). However, in this study, the large mass showed mixed hyperintensity on T2WI and FLAIR, and an enhancement effect was noted only around the mass and close to the meninges. In a previous report of a dog, the intracranial MPNST containing necrotic tissue showed an enhancement of the mass margin, with no enhancement of the necrotic area on MRI (6). Our patient may have a similar pathology, but the necrotic area of the tumor was not detected during the histopathological examination. This factor includes not enough samples collected at biopsy. Additionally, intracranial and extracranial lesion formation, with occipital bone erosion, indicated that the tumor was extremely aggressive. These findings differed from those MPNSTs of cranial nerve origin and supported the aggressive nature of the tumor. Therefore, the tumor was presumed to be a subcutaneous MPNST rather than a cranial nerve origin.

Depending on the site of origin, MPNST treatment includes local therapy, such as surgical resection (2–4, 13, 27), radiation therapy (28, 29), or palliative therapy. However, a radical cure cannot be expected owing to the high local recurrence rate. To date, no effective chemotherapy has been established. In this study, complete surgical resection was expected to be difficult. Additionally, the owner did not want radiation therapy. Eventually, palliative treatment was chosen at the owner's request. However, the patient experienced a rapid decline in his condition and died 19 days after the onset of symptoms with no obvious improvement. The lesion's proximity to the brainstem and increased intracranial pressure are considered crucial factors indicating poor prognosis. In our case, urgent surgical resection for decompression could have prevented further deterioration of the condition and improved short-term prognosis.

Neoplastic diseases affecting the CNS commonly develop in middle and advanced ages and have a subacute or chronic progressive course. Since our patient developed acute paradoxical vestibular disorder at an advanced age, we considered vascular or inflammatory diseases rather than neoplastic diseases as the major differential diagnosis at the time of onset. Brain edema associated with intracranial invasion of the tumor might have caused the acute onset of vestibular disorder. Subsequently, progressively worsening clinical signs after onset were an important finding that suggested a neoplastic disease. Therefore, it is crucial to always consider the possibility of neoplastic diseases and evaluate the subsequent disease course, even when acute vestibular disorder occurs in older animals.

A limitation of this case report is that autopsy could not be performed because the owner's consent was not obtained. From this reason, the exact origin of the tumor and the route of intracranial invasion could not be determined. Furthermore, the small sample size of tumor did not allow for sufficient immunostaining to make a more accurate diagnosis. Hence, in order to make an accurate diagnosis, it is important to collect samples from a number of locations and to be prepared to perform various immunochemical staining as needed.

## Data availability statement

The original contributions presented in the study are included in the article/supplementary material, further inquiries can be directed to the corresponding author.

## Ethics statement

Ethical review and approval was not required for the animal study because ethical review and approval are not required for retrospective case reports. Written informed consent was obtained from the patient's owner for publication of this report. Written informed consent was obtained from the owners for the participation of their animals in this study.

## Author contributions

KH, KS, YS, KN, MN, and YN assisted with the diagnosis of this case and participated in clinical case management. KH and YN participated in the review and editing of the manuscript. All authors contributed to the article and approved the submitted version.

## Conflict of interest

Author KN was employed by FUJIFUILM VET Systems Co., Ltd. The remaining authors declare that the research was conducted in the absence of any commercial or financial relationships that could be construed as a potential conflict of interest.

## Publisher's note

All claims expressed in this article are solely those of the authors and do not necessarily represent those of their affiliated organizations, or those of the publisher, the editors and the reviewers. Any product that may be evaluated in this article, or claim that may be made by its manufacturer, is not guaranteed or endorsed by the publisher.
